# Measurement and compensation of misalignment in double-sided hard X-ray Fresnel zone plates

**DOI:** 10.1107/S1600577520001757

**Published:** 2020-03-18

**Authors:** Viktoria Yurgens, Frieder Koch, Mario Scheel, Timm Weitkamp, Christian David

**Affiliations:** a Paul Scherrer Institute, 5232 Villigen PSI, Switzerland; b Synchrotron SOLEIL, 91192 Gif-sur-Yvette, France

**Keywords:** X-ray optics, Fresnel zone plate, diffraction efficiency, microfocus X-ray source

## Abstract

Double-sided Fresnel zone plates are diffractive lenses used for high-resolution hard X-ray microscopy. The double-sided structures have significantly higher aspect ratios compared with single-sided components and hence enable more efficient imaging. The zone plates discussed in this paper are fabricated on each side of a thin support membrane, and the alignment of the zone plates with respect to each other is critical. Here, a simple and reliable way of quantifying misalignments by recording efficiency maps and measuring the absolute diffraction efficiency of the zone plates as a function of tilting angle in two directions is presented. The measurements are performed in a setup based on a tungsten-anode microfocus X-ray tube, providing an X-ray energy of 8.4 keV through differential measurements with a Cu and an Ni filter. This study investigates the sources of the misalignments and concludes that they can be avoided by decreasing the structure heights on both sides of the membrane and by pre-programming size differences between the front- and back-side zone plates.

## Abstract 2

A simple and reliable way of characterizing double-sided hard X-ray Fresnel zone plates is presented. The measurements are performed in an X-ray tube-based setup where a near-monochromatic spectrum is isolated by differential measurements with a Cu and an Ni filter.

## 1. Introduction   

Fresnel zone plates (FZPs) are diffractive lenses used as focusing elements in a range of soft and hard X-ray imaging applications in synchrotrons all over the world. In their most common form, they are circular gratings consisting of a series of concentric rings, or ‘zones’, with gradually decreasing widths towards the periphery. The zones are spaced such that light transmitted through the zone plate interferes constructively at an intended focal distance. The interference occurs due to a relative phase or amplitude difference induced in beams emerging from neighbouring zones (CXRO; Kirz, 1974 ▸).

The diffraction efficiency of a zone plate, defined as the fraction of the incoming intensity diffracted into a certain diffraction order, is one of its most important characteristics (Buralli & Morris, 1992 ▸). The maximum diffraction efficiency is reached when the zone plate structure introduces a phase shift close to π to the incoming X-ray wave. For hard X-rays, this requires that the zones must be several micrometres thick, even when the zone plate is fabricated out of heavy materials (Snigirev & Snigireva, 2008 ▸). At the same time, in order to be able to perform high-resolution imaging, the outermost zone width *dr* needs to be very small, in many cases down to tens of nanometres. The resulting extreme aspect ratios are very difficult to fabricate.

A way of overcoming the aspect ratio limitations in the fabrication is to stack two or more zone plates on top of each other, so that they work as a single zone plate with a zone height equal to the total height of the structures (Maser *et al.*, 2002 ▸; Snigireva *et al.*, 2007 ▸; Gleber *et al.*, 2014 ▸). The approach bears the challenge of maintaining the mechanical alignment of the stacked zone plates during operation within a fraction of the outermost zone width. This problem can be avoided by making double-sided zone plates (Mohacsi *et al.*, 2015 ▸), where the zone plates are fabricated as a monolithic device, on each side of a thin support membrane. An additional approach to achieving small linewidths at high aspect ratios is provided by a technique called line-doubling (Jefimovs *et al.*, 2007 ▸; Vila-Comamala *et al.*, 2011 ▸). In this method, template structures are fabricated from a low-atomic-number material, *e.g.* hydrogen silsesquioxane (HSQ), which has a very small effect on the transmission of hard X-rays. These structures are then coated with a heavy metal, for example Ir, by atomic layer deposition (ALD). The interaction with X-rays is then dominated by the metal deposited on the side-walls of the template structures, which double the effective number of lines.

**Figure 1:**
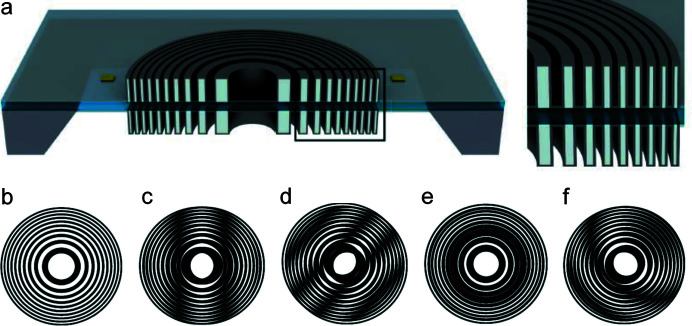
(*a*) Schematic of a double-sided line-doubled Fresnel zone plate. The HSQ template structures are indicated in light grey and the Ir deposited on top is marked in dark grey. Adapted from Mohacsi *et al.* (2015 ▸). (*b*) Sketch of a Fresnel zone plate. (*c*)–(*f*) Different ways of overlaying two zone plates such as the one in (*b*), illustrating various types of moiré fringes.

Fig. 1 ▸(*a*) shows a schematic of a double-sided and line-doubled structure. Together, these two methods make it possible to fabricate FZPs with much higher aspect ratios compared with what is possible for single-sided zone plates. However, one needs to ensure that the alignment between the two zone plates is within a fraction of the outermost zone width, since larger misalignments will impair the optical performance. In regions on a double-sided zone plate where the two sides have a radial displacement by one zone width, the local efficiency will vanish.

If strong misalignments are present, the efficiency distribution over the zone plate area – here referred to as the efficiency map – will contain moiré fringes. Such fringes are visual artefacts arising from the interference in the overlay of ordered patterns, in our case the two zone plates. The fringes can be used to determine the relative offsets and distortions of the two zone plates (Vladimirsky, 1988 ▸).

Figs. 1 ▸(*c*)–1(*f*) show how different moiré interference patterns emerge from the overlay of two zone plate sketches such as the one in Fig. 1 ▸(*b*). Figs. 1 ▸(*c*) and 1 ▸(*d*) show examples of linear offsets in different directions: in the former, the zone plates are offset with respect to each other by 2*dr* in the horizontal direction, and in the latter by 4*dr* along the diagonal. In Fig. 1 ▸(*e*), the zone plates are concentric, but there is a scaling difference of 2*dr* between the zone plate radii, resulting in a concentric circular fringe. Curved and non-centric fringes are visible in Fig. 1 ▸(*f*), where the zone plates have a scaling difference of 2*dr* and are offset by 2*dr* along the diagonal. This illustrates that straight fringes result from offsets between the zone plates, whereas curved fringes always indicate the presence of a scaling difference.

In this paper, we present an X-ray tube-based setup that yields absolute measurements of first-order diffraction efficiency of Fresnel zone plates, as well as maps of the zone plate diffraction efficiency. The setup is used to measure misalignments, to find ways to compensate for them in the fabrication and, finally, to minimize their effect in finished zone plates. The step to a laboratory-based setup is crucial to provide quick feedback to the fabrication process that would be impossible to obtain on a regular basis at a synchrotron.

## 2. Zone plate fabrication   

Arrays of double-sided, line-doubled zone plates were produced on 250 nm-thick silicon nitride membranes. The zone plates were designed with a 100 µm diameter and an outermost zone width of 50 nm to yield a focal length of 34 mm at an X-ray energy of 8.4 keV. The structures were made from an HSQ template covered with Ir by plasma-enhanced ALD; the width of the HSQ template structures as well as the Ir layer thickness were designed for optimum diffraction efficiency (Marschall *et al.*, 2017 ▸). The complete processing, including the patterning of a set of alignment markers around each zone plate, was performed first on the back-side of the membranes. Subsequently, the same processes were repeated on the front-side, using the markers on the back-side for alignment.

The chips were spin-coated with undiluted HSQ FOx-16 resist at 1000 rpm for 60 s, resulting in approximately 600 nm-thick HSQ layers on the back-side of the membrane and between 800 nm and 2 µm-thick layers on the front-side. A low rotation speed was used in order to obtain HSQ layers that were as thick as possible. The difference in height at the same rotation speed originates from the different structures of the silicon chip on its two sides due to the etching of the membrane. On the back-side, the etched side-walls of the carrier wafer affect the resist spread during spin-coating, causing a strong height gradient over about 400 µm, which is unusable for structuring. Only the central part of the membrane can therefore be used for the zone plate fabrication on the back-side. The tension due to the curved surface of the resist flowing over the sidewall is also likely to be responsible for the lower overall height on the back-side compared with the front when using identical spinning parameters. The layer thicknesses were measured on finished Ir-coated structures in a scanning electron microscope (SEM) under a 45° tilting angle.

Patterning of the HSQ layers was performed using a Vistec EBPG 5000+ electron-beam lithography system, operated at an acceleration voltage of 100 kV, a beam current of 4 nA and a dose of 15 000 µC cm^−2^. The development was carried out in a 1:3 mixture of the developer AZ A351 with water for a duration of 15 min; the structures were rinsed in water and subsequently dried by critical-point drying in a Leica EM CPD300 system. The Ir deposition was achieved using a Picosun R200 ALD tool.

## 3. X-ray tube setup design   

**Figure 2:**
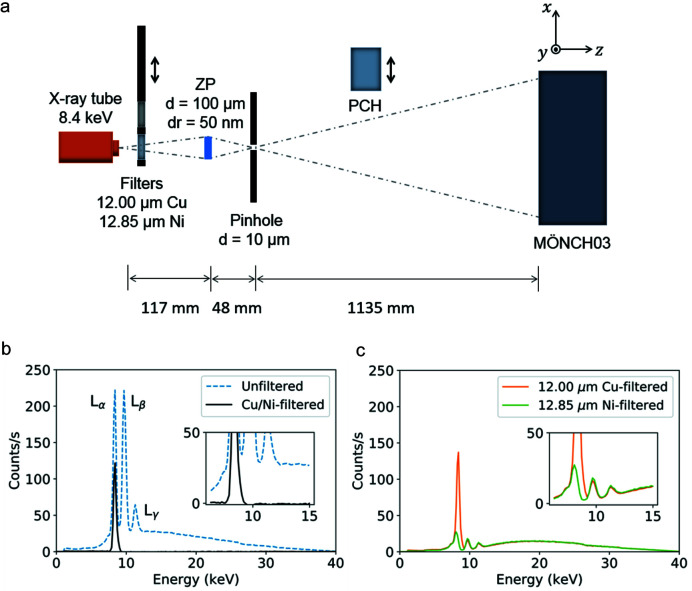
(*a*) Schematic of the X-ray tube-based setup. (*b*) Measured tungsten X-ray tube spectrum without filtering (blue, dashed) and after differential filtering with a 12 µm Cu and a 12.85 µm Ni filter (black). The main emission lines of tungsten are indicated; after filtering, only the *L*α_1,2_ line with an approximate X-ray energy of 8.4 keV remains. The separate sublines cannot be resolved by the spectrometer. (*c*) Measured tungsten X-ray tube spectrum when filtered with a 12 µm Cu filter (orange) and a 12.85 µm Ni filter (green). Subtracting the two spectra yields the final spectrum in (*b*). The insets show a closer view of the region of interest for the filtering, where it is apparent that the filter thicknesses have been matched so that the curves overlap for energies outside the *L*α_1,2_ line.

The setup, schematically illustrated in Fig. 2 ▸(*a*), was based on a Hamamatsu L10101 microfocus X-ray tube with a tungsten anode operated at 40 kV acceleration voltage and 150 µA current, resulting in a spot size of approximately 10 µm. A close-to-monochromatic beam was isolated from its spectrum by performing measurements first with a 12 µm Cu filter, then with a 12.85 µm Ni filter, and subtracting the signals [see Figs. 2 ▸(*b*) and 2 ▸(*c*)]. The filter thicknesses were chosen to match the absorption of Cu and Ni outside the transmitted window of energies. In the resulting effective spectrum, only the tungsten *L*α_1_ and *L*α_2_ line doublet remained, with X-ray energies of 8.398 keV and 8.335 keV and a relative intensity ratio of 9:1 (CXRO, Center For X-ray Optics; http://zoneplate.lbl.gov/theory). The two-filter scheme is similar to the one employed by Chen *et al.* (2008 ▸), which filters out the same tungsten lines by using a Co and a Cu filter. Our scheme yielded almost half the bandwidth in comparison due to the smaller difference between the absorption edge energies of Cu and Ni, and therefore isolated the lines of interest more effectively. The resulting monochromaticity was Δ*E*/*E* = 7.5 × 10^−3^. The filtered beam illuminated the zone plate placed 117 mm downstream of the source, and the first diffraction order was isolated using a 10 µm pinhole placed in the focal plane 48 mm downstream of the zone plate. The emerging light was projected onto a MÖNCH03 direct-conversion hybrid Si pixel detector (Ramilli *et al.*, 2017 ▸; Bergamaschi *et al.*, 2018 ▸) placed 1135 mm downstream from the pinhole and yielding a projection of the efficiency distribution of the zone plate. In the diffraction efficiency measurements, a Hamamatsu Photon Counting Head H10682-210 (PCH) was placed behind the pinhole and counted the transmitted flux. For the conversion of X-rays to visible photons, a 5.5 mm-thick polyvinyl toluene scintillator (BC400, Saint-Gobain) was used.

**Figure 3:**
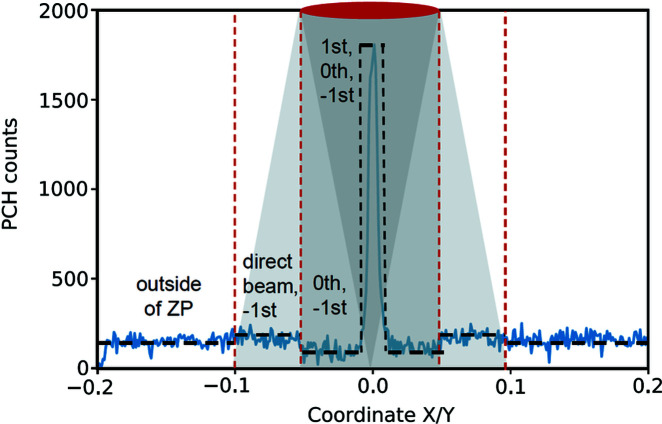
Schematic of the counts recorded on the PCH as a function of zone plate position along one of the scanning directions. Shaded regions in the background illustrate how the zone plate (in red) diffracts the incoming light into the distinct diffraction orders. The red dashed line indicates the resulting regions with different count levels within and outside of the zone plate.

The efficiency measurements were performed by scanning the zone plate in a plane perpendicular to the optical axis and recording the photons diffracted into the first order with the PCH. Scans were performed with a step length of 1 µm over a range covering three times the zone plate diameter; the counts were integrated for 10 s at each point. Line-doubled zone plates suffer from an increased contribution of the zeroth order in the centre, which makes it difficult to determine the number of first-order counts in the peak in the resulting scans. However, since the central part of the zone plate mainly consists of low-*Z* HSQ, the zeroth-order counts at that position can be well approximated by the number of photons passing through the silicon nitride membrane outside of the zone plate. This is why a broad scanning range was chosen (see Fig. 3 ▸). By subtracting the number of counts in the region labelled ‘outside of ZP’ from the number of counts in the region labelled ‘1st, 0th, −1st’, one is effectively subtracting the slightly elevated number of photons diffracted into the zeroth order in the centre of the zone plate as well as the counts in the negative first order, and obtaining the first order counts. Reference measurements to account for the incoming photons were made by removing the 10 µm pinhole and replacing the zone plate by a 100 µm pinhole. All measurements were differential, meaning that they were performed once with the Cu filter and once with the Ni filter, with the final result given after subtraction of the two signals. Dividing the first-order counts by the number of counts in the reference measurements finally gave the efficiency of the zone plate.

## 4. Results   

**Figure 4:**
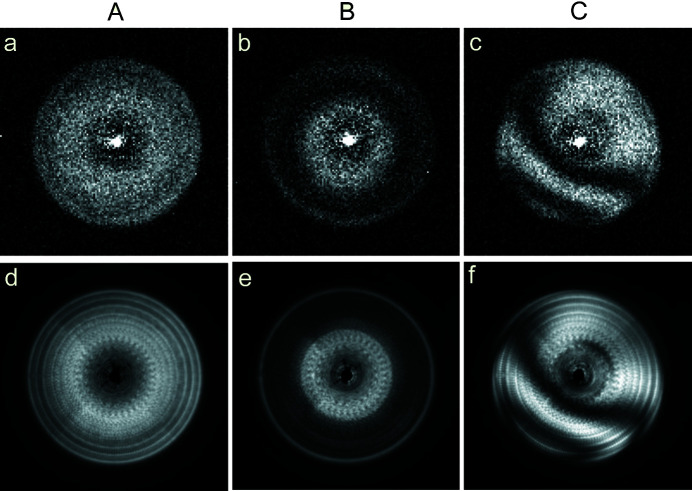
(*a*)–(*c*) Diffraction efficiency maps recorded with the X-ray tube setup at an X-ray energy of 8.4 keV and an exposure time of 1400 s. (*d*)–(*f*) Diffraction efficiency maps of the same three zone plates made at the ANATOMIX beamline of Synchrotron SOLEIL at an X-ray energy of 10 keV with an exposure time of 2 s. The zeroth-order counts in the centre of the maps have been subtracted.

Fig. 4 ▸ shows a comparison between imaging performed with the X-ray tube and at a synchrotron beamline. Figs. 4 ▸(*a*)–4(*c*) show normalized diffraction efficiency maps of three double-sided line-doubled zone plates from the same chip labelled A, B and C, recorded in the X-ray tube setup at an energy of 8.4 keV with a 1400 s exposure time. The drift in the relative position of the pinhole and zone plates was negligible, enabling prolonged exposure times. Figs. 4 ▸(*d*)–4(*f*) show normalized maps made of the same zone plates at the ANATOMIX beamline of Synchrotron SOLEIL (Weitkamp *et al.*, 2017 ▸; Scheel *et al.*, 2018 ▸) at an X-ray energy of 10 keV with a 2 s exposure time. The measurement geometry at the beamline was similar to that presented in Fig. 2 ▸, but with a parallel beam impinging on the zone plate and a 25 µm pinhole instead of the smaller one used with the laboratory setup, with an indirect scintillator-coupled detector (pco.4000 with 1× detector optics) 4 m downstream of the zone plate. The synchrotron images show higher resolution, revealing subtle details in the efficiency maps and a signal-to-noise ratio 6.5 times higher than the X-ray tube images, but the important information about the alignment given by the moiré patterns is clearly also obtained from the maps made with an X-ray tube as the source.

The efficiencies obtained for zone plates A, B and C at 8.4 keV were 5.6%, 3.0% and 5.3%, respectively. The large differences between the zone plate efficiencies can be explained by the moiré patterns in the three cases, showing that A is a double-sided zone plate with little misalignment, B is a double-sided zone plate with good alignment but a scaling difference between the zone plate on the front- and back-side, and C is a double-sided zone plate with both a size difference and an offset between its halves. The comparably high efficiency of C can be explained by its total structure height being close to ideal for the measured energy.

These zone plates, made on the same chip with the same design parameters, showed vastly different alignment results. The only substantial difference between zone plates on the same chip is height, since the slow HSQ spin-coating leads to strong variations in the layer thickness over the membrane. General fabrication trends were seen where zone plates showing curved moiré fringes like C or strong concentric fringes like B had front-side zone plate heights of approximately 1.4 µm, whereas zone plates with a good alignment like A had front-side heights of only 900 nm, all compared with back-side heights of approximately 600 nm. This indicated a structure–height-related alignment and scaling problem. In the case of the scaling problem, the question arose whether the front- or the back-side zone plates had a larger diameter than their counterparts.

As a test of the influence of the height differences, and to determine the sign of the scaling error, two double-sided chips were prepared with identical layouts but different front-side structure heights. The layouts consisted of a three-by-three grid of zone plates, all identical on the back-side with a 100 µm diameter and 50 nm outermost zone width, but different on the front-side where the zone plate diameters were varied from −0.5% to +0.5% in one direction and diagonal offsets in steps of 100 nm were introduced in the other. The chips were spin-coated at 2500 rpm on the back-side, giving structure heights of 550 nm, and at 1500 rpm and 2500 rpm on the front-side, giving structure heights of 1200 nm (chip 1) and 750 nm (chip 2), respectively.

**Figure 5:**
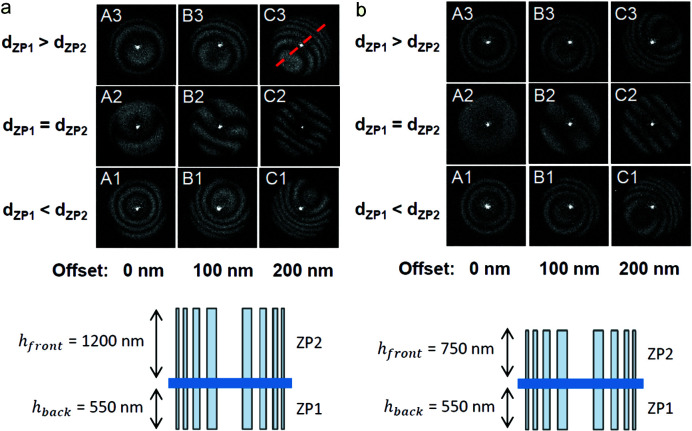
(*a*) Diffraction efficiency maps of the zone plates on chip 1 with a large difference in height between the back-side zone plate ZP1 and the front-side zone plate ZP2. (*b*) Diffraction efficiency maps of the zone plates on chip 2 with a small difference in height between the front- and back-side zone plates. The relative sizes (diameters) of the zone plates on the back-side and front-side are varied along the vertical axis in the grids and the diagonal offsets between them are varied along the horizontal axis. The red dashed line indicates the direction along which the offsets are made.

Fig. 5 ▸(*a*) shows the efficiency maps of the zone plates on chip 1, the sample with a large height difference between the front- and the back-side structures. These and all of the following diffraction efficiency maps presented in this paper were made at the X-ray tube setup with a total acquisition time of 700 s. The case with no intentional offset or size difference, A2, shows the least fringes. The maps confirm the expected trends; the number of fringes increases with the offset, and their direction corresponds to the diagonal offset. By counting the number of dark moiré fringes in column A – zone plate A1 has three, A2 has one and A3 has two but with a shifted centre – one can conclude, by simple interpolation, that a case between the latter two, with the back-side zone plate being 0.2% larger in diameter than the front-side, would compensate for the size difference between the zone plates.

Fig. 5 ▸(*b*) shows the efficiency maps of chip 2, the sample with a small height difference between the front- and back-side structures. The alignment is generally much better than for chip 1 and there are no indications of scaling issues in row 2 where no intentional size differences have been made. In column A, the moiré fringes are purely concentric, indicating a good alignment. Zone plates A1 and A3 are symmetric around zone plate A2 in terms of the number of fringes, indicating that A2 is the best case and showing that the optimal double-sided zone plate for these structure heights is obtained when the front- and back-side zone plates are fabricated to be the same size.

We can conclude that it is more difficult to guarantee a good alignment between the parts of a double-sided zone plate when the structure heights on the front-side are much higher than on the back-side. When such a case arises, one must find ways to compensate for the offset issues appearing, which is difficult since these typically vary for every zone plate. In order to compensate for the scaling issues for a sample such as chip 1 with an average front-side structure height of 1.2 µm and a back-side structure height of 550 nm, the zone plates on the back-side should be designed to be 0.2% larger in diameter than the corresponding front-side zone plates. When the structure heights on the back-side and front-side are more similar, such as on chip 2, no compensation is needed.

A possible origin of the scaling and alignment issues is the shrinking of the HSQ after the electron beam exposure, possibly induced by the Ir deposition. Since the Ir is deposited on the back-side structures before the frontside patterning, the elevated temperatures in the process could lead to a decrease in size which subsequently affects the front-side alignment. The larger the difference in height between the zone plates on the two sides of the membrane, the stronger the effect. We noted that the electron beam system measured smaller distances between the alignment markers on the back-side compared with what was written, and correspondingly rescaled the structure on the front-side before writing it. Due to the somewhat different measured positions of the markers, offsets were likewise introduced. In addition, since the system uses three markers for the alignment, it obtains one scaling factor for each of the two directions spanning the membrane plane, meaning that in some cases even slightly elliptical zone plates were patterned on the front-side. Possible changes to the alignment algorithm of the electron beam system are being investigated.

**Figure 6:**
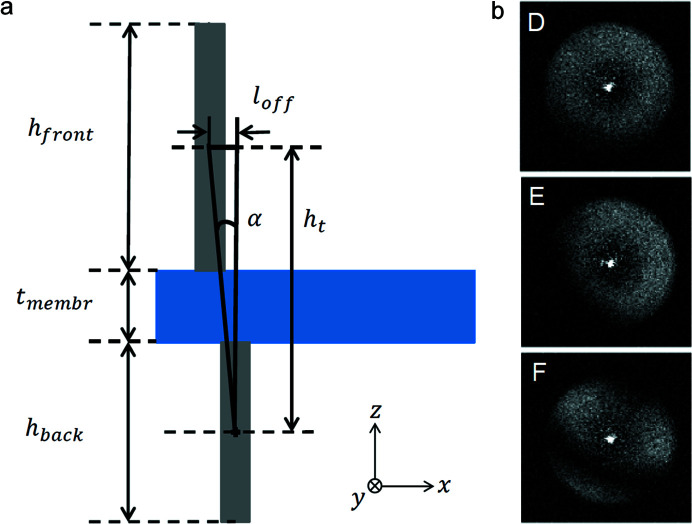
(*a*) Schematic of a front-side zone offset a distance *l*_off_ with respect to the corresponding back-side zone. Tilting angle α will realign the centres of mass of the two zones and should maximize the diffraction efficiency of the total structure. (*b*) Zone plates with different degrees of misalignment, chosen for the tilting measurements. The diagonal fringes on zone plate F indicate a strong misalignment along both the *x* and the *y* directions.

The misalignments in double-sided structures were studied further by means of zone plate tilting. Fig. 6 ▸(*a*) illustrates that, for small offsets of the front-side zone plate by a distance *l*_off_ with regard to the back-side zone plate, there is a tilting angle α for which the centres of mass of the zones on the opposing sides of the membrane are aligned again. Such an angle should maximize the efficiency, compensate for the misalignment and remove the moiré fringes in the corresponding direction. Through simple geometrical considerations with the thickness of the silicon nitride membrane *t*_membr_, the heights on the back-side and front-side *h*_back_ and *h*_front_, and the optimal tilting angle α, we can calculate the offset from *l*_off_ = (*h*_front_/2 + *t*_membr_ + *h*_back_/2)tanα. Fig. 6 ▸(*b*) shows diffraction efficiency maps of three zone plates D, E and F, on which these measurements were performed. The structures were spin-coated at a speed of 2500 rpm on both the back-side and the front-side, giving structure heights of 550 nm and 750 nm, respectively, and a specific set of zone plates was chosen to represent different degrees of misalignment.

**Figure 7:**
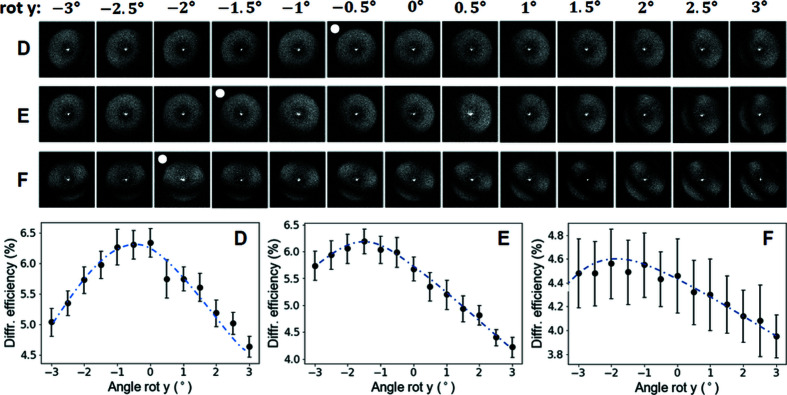
Diffraction efficiency maps of three different zone plates for different rotation angles around the *y* axis (top) and the corresponding diffraction efficiencies (bottom). White dots mark the maps where the moiré fringes in the *x* direction are minimized; the optimal angles correspond well with the maxima of the approximate fits made in the efficiency curves.

In order to determine the misalignment in the double-sided zone plates along the *x* direction, they were rotated around the *y* axis. The rotation angle around the *x* axis was kept at 0°. The angle range −3° to 3° was chosen according to predictions made from the moiré patterns in the diffraction efficiency maps at zero rotation and known front- and back-side zone plate heights. The resulting images and efficiencies are presented in Fig. 7 ▸. As expected, all three zone plates show the highest diffraction efficiency at the rotation angle with the least and best centred moiré fringes. This angle and the known structure height yield an *x*-offset for zone plates D, E and F of 8 nm, 25 nm and 33 nm, respectively. Using the optimal angle around *y* and rotating around *x*, the same procedure yielded a *y*-offset of 17 nm, 17 nm and 66 nm for the three zone plates.

The calculated offsets in *x* and *y* correspond well to the diffraction efficiency maps taken at 0° tilt angle. Two full-fringe periods over the aperture correspond to an offset of 100 nm, and in the case of zone plate F with a calculated misalignment in *x* of 33 nm and in *y* of 66 nm, we see slightly less than one period in *x* and more than one in *y*. The other zone plates show fewer fringes and correspondingly less misalignment. As a consequence of optimal tilting, the diffraction efficiencies of the zone plates increase; the increase in efficiency is small for zone plate D with the best alignment (6.3% to 6.4%), but is more substantial for zone plates E and F with larger misalignments (B: 5.7% to 6.4%; C: 4.5% to 5.9%). Consequently, a well aligned zone plate will suffer performance loss when not carefully aligned perpendicular to the optical axis.

**Figure 8:**
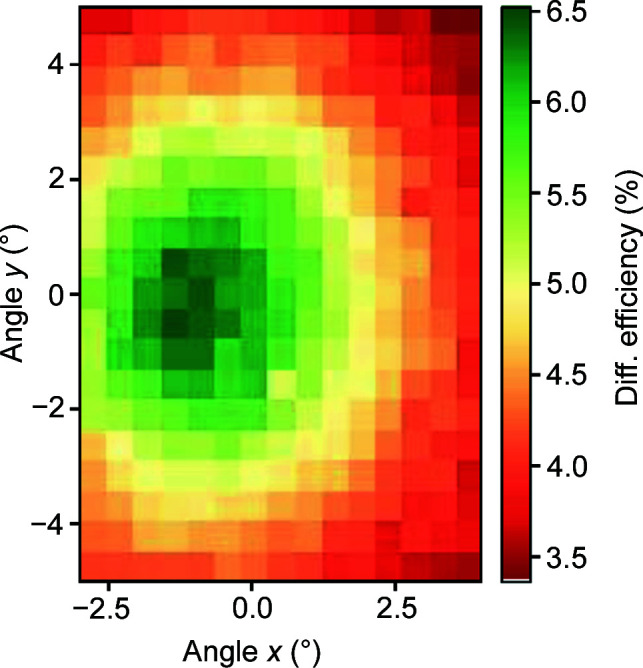
Zone plate diffraction efficiency as a function of tilting angle around *x* and *y*.

As a concluding measurement, a mesh-like scan was performed on zone plate D, where the diffraction efficiency was recorded for a range of tilting angles in *x* and *y* simultaneously. The result is presented in Fig. 8 ▸. There is a clear peak in the two-dimensional plot, centred around (rot *x*, rot *y*) = (−1°, −0.5° ), corresponding well to the optimal values found in the previous measurements. This type of scan gives a more exact result for the optimal tilting position of a zone plate but is also more time-consuming.

An advantage of the combination of tilting and efficiency mapping is the fact that one can distinguish between issues in the fabrication and issues in the alignment. A dark region in a diffraction efficiency map could indicate a fabrication problem such as undeveloped or destroyed zones or zones with an incorrect Ir duty cycle, but could also be a moiré fringe. The two cases will behave differently under tilting – the former will stay even when rotating the zone plate, whereas the latter will change just as described for the tilting tests.

Another obvious advantage of zone plate tilting is that the moiré fringes are reduced; zone plates with visible fringes produce overlapping twin images and are thus not usable for imaging. However, we need to discuss how the tilting of a zone plate affects its function as a diffractive lens in an imaging application: a tilted zone plate will appear elliptical as seen from the sample, giving a difference between the focal lengths along the minor and the major axes of this ellipse and causing astigmatism. This effect is negligible as long as the difference in focal length is well within the depth of field (DOF), given by DOF = ±2(*dr*)^2^/λ (Thomson *et al.*, 2009 ▸). In the case of our zone plate, the DOF is 1/1000 of the focal length, allowing for a relative difference between the ellipse axes of 1/2000. This limit is reached at a tilt angle of 1.8°. While this value is large enough to allow for unperturbed optical performance at the optimum tilt angles displayed in Fig. 8 ▸, it should be pointed out that zone plates with smaller *dr* or zone plates used at a lower photon energy can have a substantially smaller DOF and therefore only tolerate smaller tilt angles.

## 5. Conclusions   

The X-ray tube-based setup provides a quantitative and absolute measure of the efficiency of hard X-ray Fresnel zone plates. The obtained diffraction efficiency maps, which are fully comparable with maps measured at a hard X-ray synchrotron beamline, present important information about the spatial distribution of the efficiency and give crucial indications of misalignment, providing insight and continuous feedback on the zone plate fabrication process. In this way, the setup provides straightforward means of quality control and study of Fresnel zone plates without relying on access to synchrotron beamlines. Using the information obtained with the setup and by tilting zone plates in two directions, a method has been found to both measure and compensate for the misalignments occurring in the fabrication of double-sided line-doubled zone plates. Within limits, the performance of zone plates initially unsuitable for imaging applications can be recovered by using them at an optimal inclination.
